# Comparative Efficacy of 2 L Polyethylene Glycol Alone or With Ascorbic Acid vs. 4 L Polyethylene Glycol for Colonoscopy: A Systematic Review and Network Meta-Analysis of 12 Randomized Controlled Trials

**DOI:** 10.3389/fmed.2019.00182

**Published:** 2019-08-21

**Authors:** Xu Tian, Bing Shi, Hui Chen, Xiao-Ling Liu, Rong-Ying Tang, Yuan-Ping Pi, Wei-Qing Chen

**Affiliations:** ^1^Chongqing Key Laboratory of Translational Research for Cancer Metastasis and Individualized Treatment, Department of Gastroenterology, Chongqing University Cancer Hospital, Chongqing Cancer Institute, Chongqing Cancer Hospital, Chongqing, China; ^2^Chongqing Key Laboratory of Translational Research for Cancer Metastasis and Individualized Treatment, Department of Nursing, Chongqing University Cancer Hospital, Chongqing Cancer Institute, Chongqing Cancer Hospital, Chongqing, China

**Keywords:** colonoscopy, bowel cleansing, polyethylene glycol, ascorbic acid, meta-analysis

## Abstract

**Background:** Colonoscopy remains an optimal approach for early detection and treatment of gastrointestinal lesions, however adequate bowel preparation is the critical contributor to effective and safe colonoscopy. Polyethylene glycol (PEG)-based bowel cleansing regime has been the first recommendation before colonoscopy, however it remains unknown which regime is the optimal option.

**Aim:** The aim of our study is to determine the comparative efficacy of 2 L PEG alone or plus ascorbic acid (Asc) vs. 4 L PEG alone for bowel cleansing prior to colonoscopy.

**Methods:** We assigned two independent investigators to search and screen potential records, extract essential information, and appraise the risk of bias of individual study accordingly. Then, we adopted RevMan 5.3, Stata 14.0, and WinBUGS 1.4 software to perform all statistical analyses. We also calculated the surface under the cumulative ranking curve (SCURA) in order to rank all regimes.

**Results:** Twelve studies involving 4,106 patients were analyzed finally. Pooled results indicated an improved bowel preparation efficacy in 2 L PEG plus ascorbic acid with split-dose regime rather than in 2 L PEG plus ascorbic acid (OR, 0.25; 95% CI, 0.18–0.36), 4 L PEG with split dose (OR, 3.18; 95% CI, 2.17–4.66), and 4 L PEG (OR, 4.53; 95% CI, 3.07–6.67) regimes, which was confirmed by network meta-analyses; a better compliance in 2 L PEG plus Asc with split dose (OR, 3.08; 95% CI, 1.51–6.30) and 4 L PEG with split dose (OR, 0.43; 95% CI, 0.22–0.82) regime rather than in 4 L PEG regime, but network meta-analyses generated inconsistency results; a higher preference in 2 L PEG plus Asc with split dose regime rather than in 4 L PEG split dose (OR, 2.24; 95% CI, 1.02–4.90), which were not supported by network meta-analyses; no statistically significant difference when all regimes compared with each other in terms of adverse events.

**Conclusions:** As for bowel preparation before colonoscopy, 2 L PEG ascorbic acid with split dose should be optimally prescribed. Further studies investigating the comparative efficacy of 2 L PEG related to 4 L PEG, 4 L PEG with split dose, and 2 L PEG plus ascorbic acid with split dose, respectively are needed.

## Introduction

Colorectal cancer (CRC) is one of the most common digestive tract malignancies around the world and is a critical contributor to morbidity and mortality resulting from cancer (1). To date, colonoscopy remains the optimal option of early detecting and preventing CRC (2). Issued data indicated a reduction of about 50% in mortality of CRC when abnormal lesions in digestive tract were early endoscopic resected (3, 4). It is noted that, however, the quality of bowel cleansing is the major contributor to efficacy and safety of colonoscopy (5). The study reported by Canard JM and colleagues revealed a severe fact that more than 40% of colonoscopy failure resulted from inadequate bowel preparation (6). It is important that inadequate bowel preparation is associated with numerous adverse consequences, such as missed detection of lesions and increased economic costs (7). The findings from epidemiological studies suggested that a great deal of factors have potential of increasing the risk of poor bowel cleansing (8), however, three of which including low patient's compliance with the recommended bowel solution, poor palatability of bowel cleansing solution, and large volume of liquid account for 20 to 25% poor bowel cleansing (7). However, low patient's compliance with bowel cleansing regime plays a greatly important role in successful colonoscopy (9).

Considering these issues, a great deal of innovative bowel cleansing regimes has been developed in order to further improve the quality of bowel cleansing prior to colonoscopy, such as several polyethylene glycol (PEG)-based bowel cleansing regimes. Nevertheless, PEG-based bowel cleansing regime remains the optimal option (10). It is noted that patients are difficult to drink 4 L PEG solution due to large volume of fluid and poor palatability (11). So, many modified regimes, including split dose, low volume (2 L), low volume combined with ascorbic acid (Asc), have been developed to increase the compliance with and acceptance to large volume PEG bowel cleansing solution (4 L) (11). Many published clinical trials compared the effects and safety of split vs. same day dose (12), low volume plus ascorbic acid vs. traditional volume (13), and low volume combined with ascorbic acid related to low volume alone (14). Whereas, study regarding low volume compared to traditional volume, low volume compared to low volume supplemented with ascorbic acid with split dose, and low volume compared to traditional volume with split dose has not yet been reported. Moreover, it is important that individual study has inadequate power of identifying subtle clinical differences due to smaller sample size (15). Although several previous systematic reviews and meta-analyses investigated the comparative efficacy and safety of low volume related to traditional volume (16), low volume combined with ascorbic acid related to traditional volume (17), and split dose related to same day dose (18, 19), they provided only fragmentary pairwise results, but no comprehensive results comparing the all regimes.

It is a fact that traditional meta-analysis cannot be used to compared the efficacy and safety of more than 2 intervention arms. So Bayesian network meta-analysis, an expansion of traditional meta-analysis, has been developed on the basis of Markov Chain Monte Carlo (MCMC) and Gibbs Sampling in order to comprehensively investigate the efficacy and safety of multiple interventions which were not directly compared in individual RCT (20). And thus, we performed the present systematic review and network meta-analysis to further investigate comparative efficacy and safety of 2 L PEG alone or supplemented with ascorbic acid compared to 4 L PEG for bowel cleansing prior to colonoscopy.

## Methods

We developed and carried out the present traditional pairwise and network meta-analysis according to the criteria of the Cochrane Handbook for Systematic Reviews of Interventions (21) and reported all accumulated results in accordance with the recommendations of the preferred reporting items for systematic review and meta-analysis for network meta-analysis (PRISMA-NMA) (22). The protocol has been registered at the PROSPRO with an identical number of CRD42017068957 (23). Moreover, the published protocol of the present study can be found from BMJ Open (24). No patients written inform consent and ethical approval were not needed because we performed all statistical analyses based on published data.

### Selection Criteria

We developed our selection criteria on the basis of aims: (i) adult patients receiving elective colonoscopy, regardless of outpatients or inpatients; (ii) PEG-based bowel cleansing regimes including 4 L PEG and 2 L PEG supplemented with ascorbic acid with same day or split dose and did not combine with other agents; (iii) bowel preparation efficacy was regarded as primary outcome, and compliance with the recommend bowel cleansing regime, preference to repeat the same cleansing regime, acceptance to cleansing regime, adverse events, and detection rate of polyps and adenomas and colorectal cancer were regarded as the secondary outcomes; (iv) only RCT was considered, however abstract with sufficient data was also included; (v) only full-text published in English- or Chinese was considered because we did not include a translator, who is well-versed in other languages, in the present study.

We will exclude a study when it met the following criteria: (i) insufficient information; (ii) duplication with poor quality and partial information; and (iii) other types of research, such as review and comments.

### Definition of Outcomes

In the present study, the bowel preparation efficacy was defined as an Ottawa score of < 5 or a Boston Bowel Preparation Scale (BBPS) score of ≥ 2 for all segments or an excellent or good bowel preparation designation on the Aronchik scale or other non-validated 3-, 4-, or 5-point scales (excellent, good, fair, poor, very poor) (24). Patient's compliance with bowel cleansing regime was defined as adherence to the recommend bowel cleansing solution or consumption of at least 75% of the recommend bowel cleansing solution. Preference to repeat the same regime, acceptance to the recommend regime and adverse events were recorded with the established questionnaire in individual study (i.e., defined by individual study). Detection rate of polyps or adenomas and detection rate of CRC was defined as the number of detecting polyps or adenomas and CRC respectively, which were proved histopathologically.

### Identification of Citations

We firstly performed electronic search to identify all records compared the efficacy and safety of 2 L PEG alone or supplemented with ascorbic acid related to 4 L PEG for bowel preparation prior to colonoscopy in several databases including PubMed, EMBASE, Cochrane Central Register of Controlled Trials (CENTRAL), and China National Knowledge Infrastructure (CNKI) from January 2000 to April 2017. The latest search was updated on December 2018. The search words including “*Colonoscopy*,” “*polyethylene glycols*,” and “*random*” were adopted to design all search strings according to the unique characteristics of each databases. After electronic search, we also identified the potential study through manually checked the bibliographies of all included studies and electronically retrieved Clinicaltrial.gov. It is noted that we only considered studies published in English- and Chinese language in the present study. All search algorithms were documented in Table S1.

### Data Extraction

Two reviewers adopted the data extraction form which was designed in our previous study (25) to extract basic information for each specific outcome measure from eligible study, such as leading author, publication year, basic and clinical characteristics of participants, sample size, bowel cleansing regimes, and outcomes. We contacted the corresponding author when we cannot obtain enough data. We also calculated the Kappa value in order to assess inter-investigator reliability. Any divergences about data extraction were solved on the basis of consensus.

### Quality Assessment of Individual Study

Two reviewers adopted the Cochrane risk of bias assessment tool (21, 26) to appraise the risk of bias based on seven domains including randomization sequence generation, allocation concealment, blinding of participants, blinding of study personnel, blinding of outcome assessors, incomplete outcome data, selective reporting and other bias. We labeled individual study as “*high risk of bias,”* “*unclear risk of bias,”* or “*low risk of bias.”* according to the evaluation criteria (21).

### Statistical Analysis

We firstly conducted traditional head to head meta-analysis based on random effect model, in which within- and between-studies heterogeneity were incorporated simultaneously, to calculate the summarized odds ratio (OR) and 95% confidence intervals (CIs) (27). In the present study, we calculated OR as odds of events occurred in group 1 divided by odds of events occurred in group 2. We adopted Chi square to qualitatively test the heterogeneity (28) and used *I*^2^ statistic to calculate the proportion of the overall variation that is attributable to between-study heterogeneity (29). A *I*^2^ ≥ 50% was considered as substantial heterogeneity (29). The quantitative analysis was performed when all included studies were homogeneous. A funnel plot will be drawn in order to detect publication bias when sufficient number of studies were analyzed (> 10) (30). The study with multiple arms was quantitatively incorporated in pairwise meta-analysis according to the specific comparison.

Following the traditional head to head meta-analysis, we performed the network meta-analysis based on random effects model in accordance with the methods introduced by Chaimani et al. (31). The initial values which were automatically generated from software were used to fit the model (32). We performed 70,000 iterations and 30,000 burn-in for each outcome in order to gain convergence. A comparison-adjusted funnel plot will be constructed in order to evaluate the small-study effects when sufficient number of eligible studies were analyzed in one pair of comparison (< 10) (33). The surface under the cumulative ranking curve (SUCRA) was calculate to rank targeted bowel cleansing regimes and a higher value corresponded to better result (34). We calculated the inconsistency factor by using the loop-specific method to assess the inconsistency (31).

All analyses were conducted by using the RevMan 5.3 (Copenhagen: The Nordic Cochrane Centre, The Cochrane Collaboration, 2013), Stata 14 (StataCorp, Texas), and WinBUGS 1.4 (imperial College School of Medicine at St. Mary's, London).

### Subgroup and Sensitivity Analyses

In case of possible important heterogeneity or inconsistency, we explored the possible sources using subgroup method. Subgroup analyses were designed for time of colonoscopy, patient sources and age. Sensitivity analyses were designed for bowel preparation quality by analyzing only studies considered being at low risk of bias.

## Results

### Study Selection

**Figure 1:**
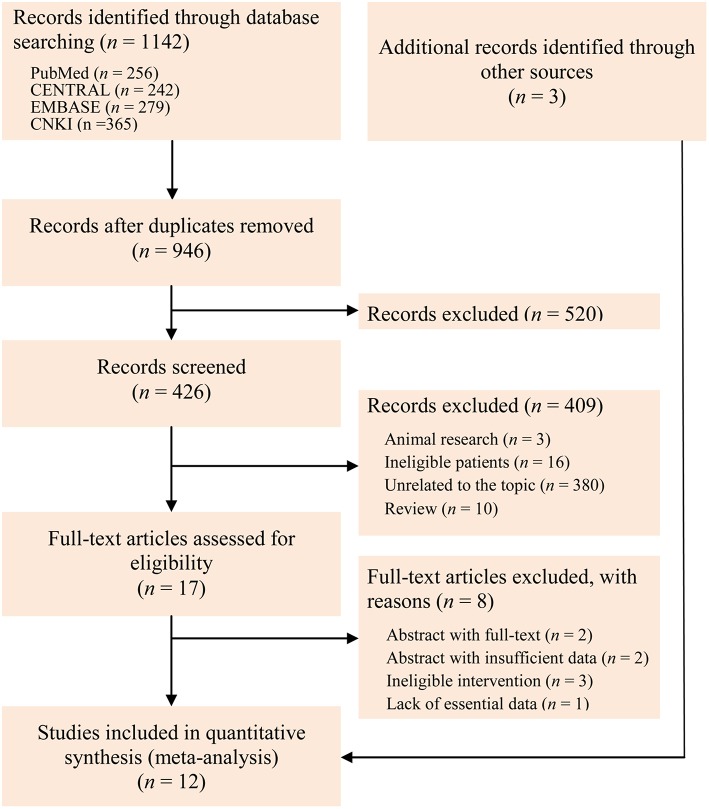
Flow chart of identification and selection of studies.

We initially identified 1,142 records through electronically searched databases. Three potential records were added through hand checked reference lists of topic-related meta-analysis. One hundred and ninety repetitive records were excluded by performing duplicate function embedded in EndNote software. After checked the title of all identified records, we excluded 520 records. And then, we checked the eligibility through screened the abstract, and 409 were excluded because of animal research, ineligible patients, unrelated to the topic, and review. Eight studies were also excluded after checked the full-text according to following reasons: abstract with published full-text, abstract with insufficient information, ineligible intervention, and lack of essential data. Twelve eligible studies (5, 11–14, 35–41) eventually incorporated into the present study. A Kappa value of 1 was reached after discussed the retrieval and selection of studies. The flow chart of identification and selection of captured studies was depicted in Figure 1.

### Study Characteristics

**Figure 2:**
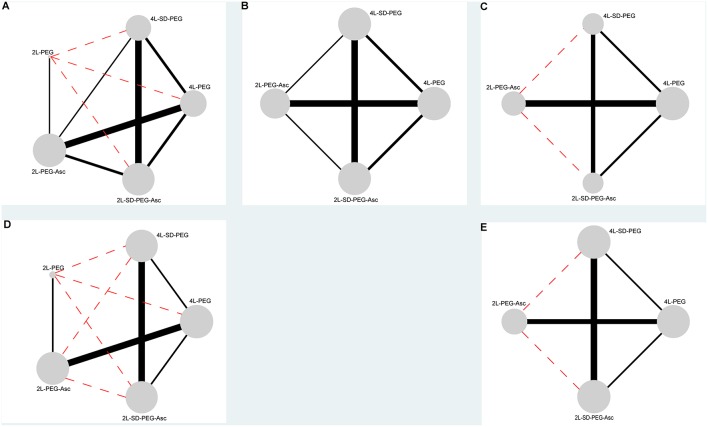
Evidence networks of all available PEG-based bowel preparation regimes in terms of **(A)** BPE, **(B)** CP, **(C)** PRSR, **(D)** AT, and **(E)** AEs. The black solid line indicated direct comparisons directly compared in original studies, and red dotted line indicated indirect comparisons which were not directly compared in original studies. The node was correspondence to total sample size and edge was proportion to precision (i.e., standard error). BPE, bowel preparation efficacy; CP, compliance with recommend regime; PRSR, preference to repeat the same regime; AT, acceptance to the regime; AEs, adverse events.

We documented the characteristics of all included studies in Table 1. Overall, the publication years were between 2008 and 2016, and most were performed in Asia. Of all 12 RCTs, 2 were the three- (35) and four-arm (5) designs respectively, and remaining 10 were two-arm design. Five different PEG-based regimes were identified including 4 L PEG with the same-day dose, 4 L PEG with split-dose, 2 L PEG with the same-day dose, 2 L PEG plus ascorbic acid, and 2 L PEG plus ascorbic acid with split-dose. The network plots of all regimes for each outcome were delineated in Figure 2. Twelve eligible studies analyzed 4,106 patients, and the actual sample size in individual study ranged from 56 to 868 with a median of 251. Of the 12 included studies, 5 (14, 36, 38–40) were the abstract with sufficient data.

**Table 1:** Basic characteristics of included studies investigating the comparative effectiveness of different PEG-based bowel preparation regimes.

**References**	**Country**	**Sample size (Male/Female)**	**Age of participants**	**Interventions**	**Outcomes**	**Randomization**
Ell et al. (11)	UK	2 L-SD-PEG-Asc: (79/74)4 L-SD-PEG: (71/84)	2 L-SD-PEG-Asc: (58.0 ± 14.7) 4 L-SD-PEG: (59.6 ± 16.0)	2 L-SD-PEG-Asc: PEG + Asc (one sachet dissolved in 1L water followed by at least 0.5L clear fluid) at each administration. 4 L-SD-PEG: PEG (two sachets dissolved in 2 L) at each administration.	BPE, CP, PRSR, AT, AEs	Computer-generated
Jung, (35)	Korea	4 L-PEG: (38/30) 4 L-SD-PEG: (33/34) 2 L-SD-PEG-Asc: (24/39)	4 L-PEG: (71.6 ± 4.7) 4 L-SD-PEG: (71.2 ± 4.4) 2 L-SD-PEG-Asc: (71.3 ± 5.0)	4 L-PEG: 4 L in the evening before the procedure (starting at 8:00 p.m.) at a rate of 250 mL every 15 min. 4 L-SD-PEG: 2 L in the evening before the procedure (starting at 8:00 p.m.) and 2 L in the morning on the day of the procedure at a rate of 250 mL every 15 min. 2 L-SD-PEG-Asc: 1L PEG with ascorbic acid followed by at least 500 mL clear fluid in the evening before the procedure (starting at 8:00 p.m.) and 1L solution followed by at least 500 mL clear fluid in the morning on the day of the procedure at a rate of 250 mL every 15 min.	BPE, CP, PRSR, AT, AEs, ADR	Computer-generated
Kanie et al. (14) §	Japan	2 L-PEG-Asc: 124 2 L-PEG: 121	2 L-PEG-Asc: 124 2 L-PEG: 121	Patients who underwent colonoscopy were randomized to ingest low volume polyethylene glycol (single dose) or polyethylene glycol with ascorbic acid solutions (single dose).	BPE, AT	Computer-generated
Kim, (12)	Korea	2 L-PEG-Asc: (85/74)4 L-PEG: (78/82)	2 L-PEG-Asc: (48.0 ± 8.8) 4 L-PEG: (45.0 ± 10.7)	2 L-PEG-Asc: 1L of 2 L-PEG-Asc at 6:00 p.m. on the day before the procedure and the remaining 1L in the morning at least 5 h prior to the procedure at a rate of 250 mL every 15 min. 4 L-PEG: 2 L PEG at 6:00 p.m. on the day before the procedure and the remaining 2 L in the morning at least 5 h before the procedure at rate of 250 mL every 15 min.	BPE, CP, PRSR, AT, AEs	Computer-generated
Lee et al. (36) §	Korea	2 L-PEG-Asc: 34 4 L-PEG: 22	57.9 (28–81)	2 L-PEG-Asc: 2 L polyethylene glycol-electrolytes with ascorbic acid. 4 L-PEG: standard 4 L polyethylene glycol-electrolytes.	BPE, CP, PRSR, AT, AEs	Computer-generated
Marmo et al. (5)	Italy	4 L-SD-PEG: (107/111) 2 L-SD-PEG-Asc: (130/87) 4 L-PEG: (117/98) 2 L-PEG-Asc: (142/76)	4 L-SD-PEG: (58.2 ± 15.9) 2 L-SD-PEG-Asc: (59.2 ± 14.8) 4 L-PEG: (57.9 ± 14.8) 2 L-PEG-Asc: (57.5 ± 13.8)	In cases of the non-split-dosage schedule, the entire dose was administered in the evening of the day before the planned colonoscopy. In cases of the split-dosage-intake schedule, half the dose was taken the afternoon before and half the dose early in the morning on the day of the colonoscopy. For the low volume solution, patients were encouraged to drink at least 1L additional clear fluid.	BPE, CP, AT, AEs, PDR	Computer-generated
Moon et al. (37)	Korea	2 L-SD-PEG-Asc: (80/83) 4 L-PEG: (84/80)	2 L-SD-PEG-Asc: (52.3 ± 11.8) 4 L-PEG: (54.0 ± 11.6)	In both groups, half the bowel-cleansing solution was administered the evening before the procedure (from 8:00 p.m.), and the remainder was administered early the morning of colonoscopy. In the 2 L-PEG-Asc arm, patients were instructed to take 1L of PEG plus Asc solution (250 mL each 15 min) followed by at least 500 mL of clear fluid at each administration. In the 4 L-PEG arm, 2 L PEG (250 mL each 15 min) was administered at each administration.	BPE, CP, AT, AEs, ADR	n.r.
Paggi et al. (38) §	Italy	2 L-SD- PEG-Asc: 335 2 L-PEG-Asc: 338	n.r.	2 L-SD-PEG-Asc: Patients who undergoing elective colonoscopy were assigned to ingest the 2 L-SD-PEG-Asc. 2 L-PEG-Asc: Patients who undergoing elective colonoscopy were assigned to ingest the standard 2 L-PEG-Asc.	BPE, ADR	Computer-generated
Park et al. (39) §	Korea	2 L-SD-PEG-Asc: 132 4 L-SD-PEG: 119	n.r.	Patients who undergoing elective colonoscopy were randomized to 2 L PEG combined with ascorbic acid or a standard 4 L PEG solutions and bowel preparations were performed with split same volume schedule in both group.	BPE, CP, AEs	n.r.
Ponchon et al. (40) §	France	2 L-PEG-Asc: (107/95) 4 L-PEG: (105/93)	2 L-PEG-Asc: (55.07 ± 12.51) 4 L-PEG: (55.93 ± 12.19)	2 L-PEG-Asc: First 1L between 6.30 and 7.30 p.m. and the second 1L between 9.00 and 10.00 p.m. 4 L-PEG: 2 L between 5.00 and 7.00 p.m. and the second 2 L between 8 and 10 p.m.	BPE, CP, AT, AEs	Computer-generated
Rivas et al. (13)	US	2 L-PEG-Asc: (64/38) 4 L-PEG: (62/42)	2 L-PEG-Asc: (57.40 ± 7.99) 4 L-PEG: (55.93 ± 7.62)	Participants in both arms were instructed to begin drinking the preparation at 6 a.m. and to finish by 10 a.m. the day of the procedure. Patients randomized to 2 L-PEG-Asc drank 16 ounces of clear liquids after each liter of the preparation as recommended by the manufacturer.	BPE, CP, PRSR, AT, AEs	Computer-generated
Valiante et al. (41)	Italy	2 L-PEG-Asc: (92/74) 4 L-PEG: (84/82)	2 L-PEG-Asc: 63 (36–82) 4 L-PEG: 65 (42–85)	2 L-PEG-Asc: 2 L from 5:00 to 8:00 p.m. (250 mL each 15 min) plus 500 mL clear fluid each liter of solution, in the evening before colonoscopy. 4 L-PEG: 2 L were administered from 3:00 to 5:00 p.m. and 2 L from 6:00 to 8:00 PM (250 mL each 15 min), in the evening before colonoscopy.	BPE, CP, AT, AEs	Computer-generated

*PEG, polyethylene glycol; Asc, ascorbic acid; BPE, bowel preparation efficacy; CP, compliance with regime; PRSR, preference to repeat the same regime; AT, acceptance to regime; AEs, adverse events; ADR, adenoma detection rate; PDR, polyp detection rate; n.r., not reported; ^§^represented abstract*.

### Quality of Individual Study

We graphically displayed the cumulative percentages for each risk of bias domain in Figure S1 and risk of bias summary for individual RCT in Figure S2. Of 12 eligible RCTs, 10 (5, 11–14, 35–38, 40, 41) appropriately generated random sequence, 3 (5, 11, 41) reported details of performed allocation concealment, 2 (12, 13) have the risk of incomplete outcome data, and all (5, 11–14, 35–41) stated in detail the process of blinded colonoscopists, reported anticipated outcomes and did not selectively reported results. Consensus principle driven a consistent judgement.

### Bowel Preparation Efficacy

All 12 eligible RCTs (5, 11–14, 35–41) reported the bowel preparation efficacy (BPE), which included seven direct-comparisons (see Figure 2A). In all seven direct-comparisons, the comparative efficacy of 2 L PEG plus ascorbic acid vs. 2 L PEG plus ascorbic acid with split dose, 2 L PEG plus ascorbic acid with split dose vs. 4 L PEG, 2 L PEG plus ascorbic acid vs. 4 L PEG with split dose, and 4 L PEG vs. 4 L PEG with split dose reached statistical significance. All pooled results from direct comparisons can be found in Figure S3.

**Figure 3:**
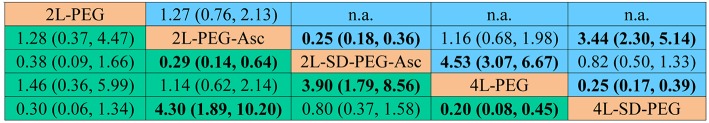
Summary for bowel preparation efficacy of different PEG-based bowel preparation regimes. The upper right area represented the effect sizes of direct comparisons and the bottom left shown the network comparisons. For direct comparison, it favors the row-defining treatment if odds ratio (OR) lower than 1, in contrast, for indirect comparison, the result favors the column-defining treatment if OR lower than 1. For numerical data, each number in each cell represented the effect size of the treatment in upper left area minus the treatment in bottom right area. Bold font represented statistical significance. PEG, polyethylene glycol; Asc, ascorbic acid; SD, split-dose; n.a., not available.

In network meta-analysis, 2 L PEG plus ascorbic acid with split dose was superior to the 2 L PEG plus ascorbic acid and 4 L PEG in terms of bowel preparation respectively, 2 L PEG plus ascorbic acid and 4 L PEG with split dose was superior to the 4 L PEG in terms of bowel preparation. Remaining comparisons were not statistically significant. All pooled results can be found in Figure 3.

**Figure 4:**
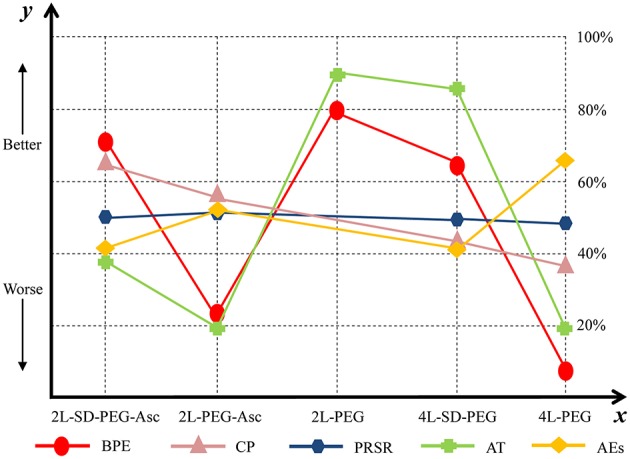
Ranking of all PEG-based bowel preparation regimes in terms of BPE, CP, PRSR, AT, and AEs. *y* axis represented a treatment will become better option from bottom to top. The percentages which were presented in right vertical dotted line represented the probability of becoming the best efficacious option and *x* axis lists all comparative nutrition support regimes. PEG, polyethylene glycol; Asc, ascorbic acid; SD, split-dose; BPE, bowel preparation efficacy; CP, compliance with recommend regime; PRSR, preference to repeat the same regime; AT, acceptance to the regime; AEs, adverse events.

We estimated SUCRA to rank all PEG-based regimes in terms of bowel preparation efficacy. The corresponding value of 2 L PEG plus ascorbic acid with split dose, 2 L PEG plus ascorbic acid, 2 L PEG, 4 L PEG with split dose, and 4 L PEG was 70.2, 22.76, 79.19, 64.6, and 13.26%, respectively. The ranking of all regimes was depicted in Figure 4.

### Compliance With Recommend Regime

Of all 12 eligible RCTs, 10 RCTs (5, 11–13, 35–37, 39–41) reported the compliance with recommend regime including six direct-comparisons (see Figure 2B). In all six direct-comparisons, the comparative efficacy of 2 L PEG plus ascorbic acid with split dose vs. 4 L PEG and 4 L PEG vs. 4 L PEG with split dose reached statistical significance. All pooled results from direct comparisons can be found in Figures S4–S8, S10.

In network meta-analysis, the pooled results from all comparisons were not statistically significant. All pooled results can be found in Figure S11A.

We estimated SUCRA to rank all PEG-based regimes in compliance with recommended regime. The corresponding value of 2 L PEG plus ascorbic acid with split dose, 2 L PEG plus ascorbic acid, 4 L PEG with split dose, and 4 L PEG was 65.8, 54.35, 36.71, and 37.25%, respectively. The ranking of all treatments was depicted in Figure 4.

### Preference to Repeat the Same Regime

All 12 eligible RCTs, 6 (11–13, 35, 36, 39) reported the preference to repeat the same regime, which included four direct-comparisons (see Figure 2C). In all seven direct-comparisons, the comparative efficacy of 2 L PEG ascorbic acid vs. 4 L PEG, 2 L PEG plus ascorbic acid with split dose vs. 4 L PEG, and 2 L PEG plus ascorbic acid with split dose vs. 4 L PEG with split dose reached statistical significance. All pooled results from direct comparisons can be found in Figures S4–S6, S8.

In network meta-analysis, the pooled results from all comparisons were not statistically significant. All pooled results can be obtained in Figure S11B.

We estimated SUCRA to rank all PEG-based regimes in preference to the same regime. The corresponding value of 2 L PEG plus ascorbic acid with split dose, 2 L PEG plus ascorbic acid, 4 L PEG with split dose, and 4 L PEG was 50.18, 50.36, 50.01, and 49.45%, respectively. The ranking of all treatments was depicted in Figure 4.

### Acceptance to the Regime

All 12 eligible RCTs, 10 (5, 11–14, 35–37, 40, 41) reported the acceptance to the regime, which included five direct-comparisons (see Figure 2D). In all five direct-comparisons, the comparative efficacy of 2 L PEG plus ascorbic acid vs. 4 L PEG and 2 L PEG plus ascorbic acid with split dose vs. 4 L PEG with split dose reached statistical significance. All pooled results from direct comparisons can be found in Figures S4–S6, S8, S9.

In network meta-analysis, 2 L PEG plus ascorbic acid with split dose was superior to the 2 L PEG plus ascorbic acid in bowel preparation respectively, 2 L PEG plus ascorbic acid and 4 L PEG with split dose was superior to the 4 L PEG in terms of bowel preparation. Remaining comparisons were not statistically significant. All pooled results can be found in Figure S11C.

We estimated SUCRA to rank all PEG-based regimes in successful bowel preparation. The corresponding value of 2 L PEG plus ascorbic acid with split dose, 2 L PEG plus ascorbic acid, 2 L PEG, 4 L PEG with ascorbic acid, and 4 L PEG was 38.75, 19.93, 87.80, 85.25, and 18.38%, respectively. The ranking of all treatments was depicted in Figure 4.

### Adverse Events

Of all 12 eligible RCTs, 10 RCTs (5, 11–13, 35–37, 39–41) investigated the adverse events, which included four direct-comparisons (see Figure 2E). In all four direct-comparisons, the comparative efficacy of all bowel preparation regimes was not statistically significant. All pooled results from direct comparisons can be found in Figures S4–S6, S8.

In network meta-analysis, 2 L PEG plus ascorbic acid with split dose was superior to the 2 L PEG plus ascorbic acid and 4 L PEG in bowel preparation respectively, 2 L PEG plus ascorbic acid and 4 L PEG with split dose was superior to the 4 L PEG in terms of bowel preparation. Remaining comparisons were not statistically significant. All pooled results can be found in Figure S11D.

We estimated SUCRA to rank all PEG-based regimes in adverse events. The corresponding value of 2 L PEG plus ascorbic acid with split dose, 2 L PEG plus ascorbic acid, 4 L PEG with split dose, and 4 L PEG was 41.80, 51.46, 41.76, and 64.97%, respectively. The ranking of all treatments was shown in Figure 4.

### Detection Rate of Polyp or/and Adenoma

Of all included studies, only three (35, 37, 38) and one (5, 38) reported the adenoma detection rate and polyp detection rate respectively, and thus we did not perform the quantitative analysis.

Jung et al. (35) found that the detection rate of adenoma was 64.7, 62.7, and 69.8% in 4 L PEG with same-day dose, 4 L PEG with split dose, 2 L PEG plus ascorbic acid with split dose group, respectively, and these differences in three comparative groups were not significant (*p* = 0.807, 0.53, 0.389, respectively). Moon et al. (37) suggested that adenoma detection rate was 43.6 vs. 41.5%, without statistical significance. Paggi et al.'s (38) study revealed an adenoma detection rate of 53.1 vs. 40.8% when 2 L PEG plus ascorbic acid with split dose compared to 4 L PEG with same-day dose, with statistical significance (*p* = 0.002).

Marmo et al. (5) performed a randomized study of split-dosage vs. non-split dosage regimens of high-volume vs. low-volume PEG solutions, and found that polyp detection rate was significantly higher in patients with bowel cleansing rated as fair/good (27.3%) or good/excellent (24.6%) compared with those with bowel cleansing rated as poor/fair (12.2%) (*p* = 0.001). The study performed by Moon and colleagues revealed that the polyp detection rate was 52.8 and 58.5% in 2 L PEG plus ascorbic acid and 4 L PEG group, however the difference was not significant.

### Detection Rate of Colorectal Cancer

All eligible studies did not report the detection rate of colorectal cancer, and thus this outcome was not statistically analyzed.

### Subgroup and Sensitivity Analyses

The present study did not perform subgroup analysis because the number of included studies for each outcome was limited. According to the protocol, we also did not carry out sensitivity analysis because of limited number of studies with low risk of bias.

### Investigation of Inconsistency

**Figure 5:**
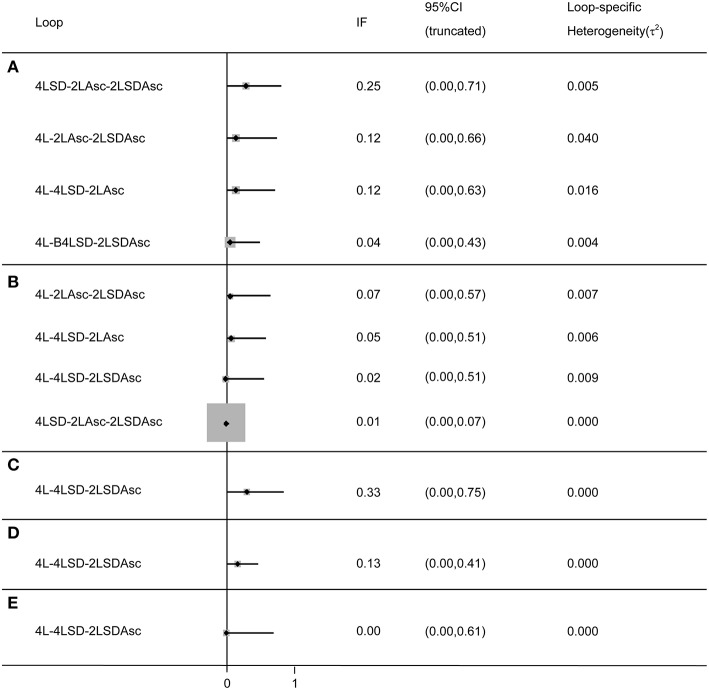
Inconsistency test for all closed loop for each outcome: **(A)** BPE, **(B)** CP, **(C)** PRSR, **(D)** AT), and **(E)** AEs. It indicates a consistency of evidences between direct and indirect comparisons if the lower limit of the 95% confidence intervals containing zero. BPE, bowel preparation efficacy; CP, compliance with recommend regime; PRSR, preference to repeat the same regime; AT, acceptance to the regime; AEs, adverse events; Asc, ascorbic acid; SD, split-dose; IF, inconsistency factor.

We adopted the split-node method to generate the inconsistency plot for the purpose of checking the consistency of results from direct and indirect comparisons. The results of inconsistency plot indicated consistency in terms of all outcomes (see Figure 5). Moreover, these results from more than 10 RCTs were pooled to comprehensively investigate the efficacy of given regimes in terms of bowel preparation efficacy, compliance with recommended regime, acceptance to the recommended regime, and adverse events, and thus, we drew comparison-adjusted funnels to test small-study effect. The comparison-adjusted funnel plots indicated asymmetrical graph for all outcomes (see Figure S12), and thus suggested that the pooled results may be negatively impacted by small study effects.

## Discussion

CRC is one of the most common gastrointestinal tract cancers, and issued data suggested that CRC is the fourth contributor to the cancer-death around the world (1). Colonoscopy remains the standard option for early prevention and detection of CRC in route practice (2). However, the diagnostic accuracy and safety of colonoscopy was mainly depending on adequate bowel cleansing (42). Although the value of many modified bowel cleansing regimes have been investigated in improving the tolerability and compliance of patients, PEG-based cleansing regime have been first-line recommendation (10). It is noted that several modified PEG-based cleansing regimes have been used in route practice, but primary study or traditional head to head meta-analysis comparing various PEG-based bowel cleansing regimes with each other has not yet been reported. And thus, it is still debate which PEG-based regime should be optimally described. So, we designed this network meta-analysis to investigate the comparative efficacy and safety of PEG-based cleansing regimes for bowel preparation prior to colonoscopy.

### Summary of Main Results

In this meta-analysis, we included 12 eligible RCTs enrolling 4,106 patients. After completed all analyses, we obtained several important findings: (i) the evidences from direct and network meta-analysis showed that 2 L PEG plus ascorbic acid with split dose and 4 L PEG with split dose obtained superior bowel preparation efficacy related to 4 L PEG, 2 L PEG plus ascorbic acid with split dose has better bowel preparation efficacy compared to 2 L PEG with split dose, and 2 L PEG plus ascorbic acid with split dose was superior to 4 L PEG with split dose in terms of bowel preparation efficacy; (ii) direct evidences suggested that 2 L PEG plus ascorbic acid with split dose has better compliance vs. 4 L PEG and 4 L PEG with split dose was better than 2 L PEG plus ascorbic acid with split dose in compliance, but these two findings were not be supported by evidences from network meta-analyses; (iii) the Preference to repeat the same regime in 2 L PEG plus ascorbic acid with split dose and 2 L PEG plus ascorbic acid were superior to that of 4 L PEG, and the 2 L PEG plus ascorbic acid with split dose was also better than 4 L PEG with split dose in terms of this given outcome, but all statistically significant findings were not detected in network meta-analyses; (iv) the direct evidence indicated improved acceptance to regime of 2 L PEG plus ascorbic acid vs. 4 L PEG and 2 L PEG plus ascorbic acid with split dose vs. 4 L PEG with split dose which were also supported by network meta-analyses, moreover, the findings of better acceptance to regime in 2 L PEG and 2 L PEG plus ascorbic acid with split dose compared to 4 L PEG and 4 L PEG with split dose respectively were determined in network meta-analyses; (v) all PEG-based regimes were comparable in terms of adverse events, which were all supported by direct and network evidences; (vi) the ranking of all PEG-based regimes was 2 L PEG, 2 L PEG plus ascorbic acid with split dose, 4 L PEG plus ascorbic acid with split dose, 2 L PEG with split dose, and 4 L PEG in terms of bowel preparation efficacy; (vii) the ranking of all PEG-based regimes was 2 L PEG plus ascorbic acid with split dose, 2 L PEG plus ascorbic acid, 4 L PEG with split dose, and 4 L PEG in improving compliance with recommended regime; (viii) for increasing preference to repeat the same regime, the ranking of all regimes was 2 L PEG plus ascorbic acid, 2 L PEG plus ascorbic acid with split dose, 4 L PEG with split dose, and 4 L PEG; (ix) for enhancing the acceptance to regime, the ranking of all PEG-based regimes was 2 L PEG, 4 L PEG with split dose, 2 L PEG plus ascorbic acid, 2 L PEG plus ascorbic acid, and 4 L PEG; (x) the ranking of all PEG-based regimes was 4 L PEG, 2 L PEG plus ascorbic acid, 2 L PEG plus ascorbic acid with split dose, and 4 L PEG with split dose for adverse events.

### Strengths and Weaknesses

Our meta-analysis has multiple strengths. Firstly, we designed comprehensive and highly sensitive search algorithms to capture any potential records and thus minimized the information bias. Secondly, our study not only analyzed direct evidence, but combined the evidences from direct and indirect comparisons, and thus more accurate estimates were generated. Thirdly, we ranked all bowel preparation regimes in terms of each outcome, which facilitates evidence-informed decision-making. Fourthly, we just included RCTs stating the word of random in analysis and abstracts with sufficient data, guaranteeing the reliability of pooled results. Fifthly, these investigators in the current study were all clinical practitioners in this given field and obtained qualification of performing a systematic review, increasing the clinical value of these findings from our study.

We also need to acknowledge a fact that some limitations existed in our study. (i) the administration time of intaking the same bowel preparation regimes and diet restriction prior to colonoscopy were slightly different from one to others, and thus our pooled results may be impaired by this bias, and thus further study should be designed to further investigate the comparative efficacy of administration times; (ii) several type of colonoscopies such as morning and afternoon were differently adopted in some eligible studies, these may affect our summarized results; (iii) although we evaluated the incidence of overall adverse events, the individual events were not investigated because of lacking of sufficient data, and the instruction value for clinical practitioners may be impaired; (iv) asymmetric comparison-adjusted funnel indicated that the robustness of the pooled results in the present study may be impaired by small study effect (25); (v) some estimates in the present study reported in individual RCT with small sample size; (vi) partial findings in the present study should be cautiously interpreted because the risk of bias may reduce the powers of some summarized results; (vii) only RCTs published in English were included, the pooled results may be impaired by selection bias; (viii) the present study did not perform the summary analysis on adenoma or/and polyp detection rate and detection rate of colorectal cancer due to limited number of included study; further study should consider these endpoint because of good bowel preparation is for better detection of lesions in clinical practice; (ix) of all included studies, only one (12) reported the objective endpoint (i.e., mucosal injury), and thus further studies should consider those objective endpoint such as mucosal injury and intestinal ecosystem.

### Agreements and Disagreements in the Current Literature

The present meta-analysis firstly investigated the comparative efficacy of various PEG-based regimes for bowel preparation prior to colonoscopy. Although most of the previous RCTs investigated the comparative efficacy individual two PEG-based bowel preparation regimes and only two simultaneously compared the potential of three and four regimes in individual study. To date, two meta-analyses with full-text have been performed to evaluate the comparative efficacy of low volume vs. traditional volume (16) and low volume plus Asc vs. traditional volume (17), and two meta-analyses which have been published in abstract investigated the efficacy of split dose vs. single dose (18, 19). However, the comparative efficacy of all available PEG-based regimes was not comprehensively in individual study. Consequently, it is unclear which PEG-based regime is optimal option in improving bowel preparation efficacy.

Godfrey et al. (16) and Xie et al. (17) separately performed traditional pairwise meta-analysis to compare 2 L PEG plus ascorbic acid with 4 L PEG regardless of usage (i.e., split- and single-dose) for BPE. Godfrey et al. (16) found no difference between 2 L PEG plus ascorbic acid and 4 L PEG in satisfactory and adverse events including abdominal pain, nausea and vomiting. The results of Xie et al. (17) indicated an improved in 2 L PEG plus ascorbic acid group for compliance with recommended regime, but no difference between these two regimes for bowel preparation efficacy. Published evidences well-demonstrated that variation in the dosage schedule may be a potential factor of bowel preparation efficacy and procedure-related complications (19). Moreover, the potential of these findings from above analyzed were limited because several PEG regimes have been used in route practice. In contrast to this, we comprehensively evaluated all available PEG-based regimes and obtained more informative findings. Firstly, we found 2 L PEG plus ascorbic acid with split dose and 2 L PEG plus ascorbic acid were associated with improved bowel preparation efficacy, compliance with recommended regime, preference to repeat the same regime, and acceptance to regime compared to 4 L PEG and 4 L PEG with split dose respectively, which consistent with previous results, but only the findings of bowel preparation efficacy and acceptance to regime were confirmed by network meta-analyses.

Avalos et al. (18) and Menard et al. (19) performed two separate meta-analyses evaluated the potential of same day compared to split-dose regime for bowel preparation before colonoscopy. Menard et al. (19) showed no difference of high-dose PEG with split dose vs. low-volume PEG with same-day dose and low-volume PEG with split dose vs. low-volume PEG with same-day dose. Avalos et al. (18) also found no difference between same-day and split dose regimes for bowel preparation efficacy, but same day regime was associated with lower incidence of adverse events. Contrary to their findings, the 2 L PEG plus ascorbic acid with split dose was better than 2 L PEG plus ascorbic acid although no statistically significant difference between 2 L PEG plus ascorbic acid with split dose and 4 L PEG with split dose in improving bowel preparation efficacy was identified. Because more eligible RCTs were included and sophisticated statistical method was adopted in the present study, and thus the results from our study were more reliable and accurate. From the view of theory, the consumption of a smaller volume of liquid and the more palatable ascorbic acid may increase the preference of patients to select the same regime in the future. We study also unfolded that 2 L PEG plus ascorbic acid with split dose and 2 L PEG plus ascorbic acid were superior to the 4 L PEG with split dose and 4 L PEG respectively for preference to repeat the same regime. Procedure-related complications are the critical factor facilitating medical decision-making. It cannot be applied into practice if the risk is more than benefit a regime can be obtained. Consequently, it is vital important to evaluate the safety before considering a regime. The adverse events were demonstrated to be similar in all regimes in our study, which was supported by the results from Godfrey et al. (16). However, Xie et al. (17) and Avalos et al. (18) generated inconsistent findings, and indicated the side effects of vomiting and nausea for 2 L PEG plus ascorbic acid were reduced relative to 4 L PEG. It is important to note that, however, individual adverse events were not explored in our study, and Xie et al. (17) and Avalos et al. (18) separately evaluated it. And thus, this variety in analysis unit may be potential reason of caused this difference. So, the further studies were warranted to investigate the impact of all PEG-based regimes on individual adverse events by using a network meta-analysis technique. In addition, the present network meta-analysis firstly makes hierarchies of different PEG-based regimes formulas including 2 L PEG plus ascorbic acid with split dose, 2 L PEG plus ascorbic acid, 2 L PEG, 4 L PEG with split dose, and 4 L PEG which were not reported in previous studies.

## Conclusions

In summary, we identified several important conclusions with significant implications for clinical practice and further research by performing this meta-analysis. Firstly, 2 L PEG plus ascorbic acid with split dose should be optimally prescribed to be bowel preparation regime prior to colonoscopy. Secondly, 2 L PEG should be further investigated because these results only generated from small numbers with small sample sizes. Further studies with large scale and well-designed were warranted because the variation in timing of bowel preparation may affect preparation quality. We also did not capture RCTs directly comparing 2 L PEG with 2 L PEG plus ascorbic acid with split dose, 4 L PEG with split dose, and 4 L PEG, and thus larger studies with excellent design are warranted.

## Data Availability

The raw data supporting the conclusions of this manuscript will be made available by the authors, without undue reservation, to any qualified researcher.

## Author Contributions

XT and W-QC conceived and designed this study. BS and HC searched and selected studies. R-YT and X-LL extracted essential information. XT and BS assessed the risk of bias. XT and Y-PP performed statistical analyses. XT and W-QC interpreted the pooled results. XT, HC, and W-QC drafted the manuscript. All authors approved the final manuscript.

### Conflict of Interest Statement

XT was employed by company TMR Integrative Nursing, TMR Publishing Group. The remaining authors declare that the research was conducted in the absence of any commercial or financial relationships that could be construed as a potential conflict of interest.
