# Supplementary material for: Comparative Efficacy of 2 L Polyethylene Glycol Alone or With Ascorbic Acid vs. 4 L Polyethylene Glycol for Colonoscopy: A Systematic Review and Network Meta-Analysis of 12 Randomized Controlled Trials
Source: Front Med (Lausanne). 2019 Aug 21;6:182. doi: 10.3389/fmed.2019.00182 (PMC6713044; doi:10.3389/fmed.2019.00182)
Supplement: Supplementary file 1 [file Table_1.pdf]

CENTRAL Search Algorithm

ID	Search
#1	Polyethylene Glycol*:ti,ab,kw or Macrogol*:ti,ab,kw or Glycol, Polyethylene:ti,ab,kw or Glycols, Polyethylene:ti,ab,kw or Polyethylene Oxide*:ti,ab,kw (Word variations have been searched)
#2	Oxide, Polyethylene:ti,ab,kw or Oxides, Polyethylene:ti,ab,kw or Polyethyleneoxide*:ti,ab,kw or Polyoxyethylene*:ti,ab,kw or Tritons:ti,ab,kw (Word variations have been searched)
#3	MeSH descriptor: [Polyethylene Glycols] explode all trees
#4	#1 or #2 or #3
#5	Colonoscop*:ti,ab,kw or Colonoscopic Surgical Procedure*:ti,ab,kw or Procedure, Colonoscopic Surgical:ti,ab,kw or Procedures, Colonoscopic Surgical:ti,ab,kw or Surgical Procedure, Colonoscopic:ti,ab,kw (Word variations have been searched)
#6	Surgery, Colonoscopic:ti,ab,kw or Surgical Procedures, Colonoscopic:ti,ab,kw or Colonoscopic Surger*:ti,ab,kw or Surgeries, Colonoscopic:ti,ab,kw (Word variations have been searched)
#7	MeSH descriptor: [Colonoscopy] explode all trees
#8	#5 or #6 or #7
#9	random*:ti,ab,kw (Word variations have been searched)
#10	MeSH descriptor: [Randomized Controlled Trial] explode all trees
#11	MeSH descriptor: [Randomized Controlled Trials as Topic] explode all trees
#12	#9 or #10 or #11
#13	#4 and #8 and #12

PubMed Search Algorithm

Search	Query
#8	Search (((("Polyethylene Glycols"[Mesh]) OR (((((((((Polyethylene Glycol*[Title/Abstract]) OR Macrogol*[Title/Abstract]) OR Glycol, Polyethylene[Title/Abstract]) OR Glycols, Polyethylene[Title/Abstract]) OR Polyethylene Oxide*[Title/Abstract]) OR Oxide, Polyethylene[Title/Abstract]) OR Oxides, Polyethylene[Title/Abstract]) OR Polyethyleneoxide*[Title/Abstract]) OR Polyoxyethylene*[Title/Abstract]) OR Tritons[Title/Abstract]))) AND (("Colonoscopy"[Mesh]) OR ((((((((Colonoscop*[Title/Abstract]) OR Colonoscopic Surgical Procedure*[Title/Abstract]) OR Procedure, Colonoscopic Surgical[Title/Abstract]) OR Procedures, Colonoscopic Surgical[Title/Abstract]) OR Surgical Procedure, Colonoscopic[Title/Abstract]) OR Surgery, Colonoscopic[Title/Abstract]) OR Surgical Procedures, Colonoscopic[Title/Abstract]) OR Colonoscopic Surger*[Title/Abstract]) OR Surgeries, Colonoscopic[Title/Abstract]))) AND random*[Title/Abstract] Sort by: PublicationDate
#7	Search random*[Title/Abstract] Sort by: PublicationDate
#6	Search ("Colonoscopy"[Mesh]) OR ((((((((Colonoscop*[Title/Abstract]) OR Colonoscopic Surgical Procedure*[Title/Abstract]) OR Procedure, Colonoscopic Surgical[Title/Abstract]) OR Procedures, Colonoscopic Surgical[Title/Abstract]) OR Surgical Procedure, Colonoscopic[Title/Abstract]) OR Surgery, Colonoscopic[Title/Abstract]) OR Surgical Procedures, Colonoscopic[Title/Abstract]) OR Colonoscopic Surger*[Title/Abstract]) OR Surgeries, Colonoscopic[Title/Abstract]) Sort by: PublicationDate
#5	Search ((((((((Colonoscop*[Title/Abstract]) OR Colonoscopic Surgical Procedure*[Title/Abstract]) OR Procedure, Colonoscopic Surgical[Title/Abstract]) OR Procedures, Colonoscopic Surgical[Title/Abstract]) OR Surgical Procedure, Colonoscopic[Title/Abstract]) OR Surgery, Colonoscopic[Title/Abstract]) OR Surgical Procedures, Colonoscopic[Title/Abstract]) OR Colonoscopic Surger*[Title/Abstract]) OR Surgeries, Colonoscopic[Title/Abstract] Sort by: PublicationDate
#4	Search "Colonoscopy"[Mesh] Sort by: PublicationDate
#3	Search ("Polyethylene Glycols"[Mesh]) OR (((((((((Polyethylene Glycol*[Title/Abstract]) OR Macrogol*[Title/Abstract]) OR Glycol, Polyethylene[Title/Abstract]) OR Glycols, Polyethylene[Title/Abstract]) OR Polyethylene Oxide*[Title/Abstract]) OR Oxide, Polyethylene[Title/Abstract]) OR Oxides, Polyethylene[Title/Abstract]) OR Polyethyleneoxide*[Title/Abstract]) OR Polyoxyethylene*[Title/Abstract]) OR Tritons[Title/Abstract]) Sort by: PublicationDate
#2	Search (((((((((Polyethylene Glycol*[Title/Abstract]) OR Macrogol*[Title/Abstract]) OR Glycol, Polyethylene[Title/Abstract]) OR Glycols, Polyethylene[Title/Abstract]) OR Polyethylene Oxide*[Title/Abstract]) OR Oxide, Polyethylene[Title/Abstract]) OR Oxides, Polyethylene[Title/Abstract]) OR Polyethyleneoxide*[Title/Abstract]) OR Polyoxyethylene*[Title/Abstract]) OR Tritons[Title/Abstract] Sort by: PublicationDate
#1	Search "Polyethylene Glycols"[Mesh] Sort by: PublicationDate

Embase Search Algorithm

No. Query

#10. polyethylene:ab,ti AND glycol*:ab,ti OR macrogol*:ab,ti OR (glycol,:ab,ti AND polyethylene:ab,ti) OR (glycols,:ab,ti AND polyethylene:ab,ti) OR (polyethylene:ab,ti AND oxide*:ab,ti) OR (oxide,:ab,ti AND polyethylene:ab,ti) OR (oxides,:ab,ti AND polyethylene:ab,ti) OR polyethyleneoxide*:ab,ti OR polyoxyethylene:ab,ti OR tritons:ab,ti OR 'macrogol derivative'/exp AND (colonoscop*:ab,ti OR (colonoscopic:ab,ti AND surgical:ab,ti AND procedure*:ab,ti) OR (procedure,:ab,ti AND colonoscopic:ab,ti AND surgical:ab,ti) OR (procedures,:ab,ti AND colonoscopic:ab,ti AND surgical:ab,ti) OR (surgical:ab,ti AND procedure,:ab,ti AND colonoscopic:ab,ti) OR (surgery,:ab,ti AND colonoscopic:ab,ti) OR (surgical:ab,ti AND procedures,:ab,ti AND colonoscopic:ab,ti) OR (colonoscopic:ab,ti AND surger*:ab,ti) OR (surgeries,:ab,ti AND colonoscopic:ab,ti) OR 'colonoscopy'/exp) AND (random*:ab,ti OR 'randomized controlled trial'/exp OR 'randomized controlled trial (topic)'/exp)

#9. random*:ab,ti OR 'randomized controlled trial'/exp OR 'randomized controlled trial (topic)'/exp

#8. 'randomized controlled trial'/exp OR 'randomized controlled trial (topic)'/exp

#7. random*:ab,ti

#6. colonoscop*:ab,ti OR (colonoscopic:ab,ti AND surgical:ab,ti AND procedure*:ab,ti) OR (procedure,:ab,ti AND colonoscopic:ab,ti AND surgical:ab,ti) OR (procedures,:ab,ti AND colonoscopic:ab,ti AND surgical:ab,ti) OR (surgical:ab,ti AND procedure,:ab,ti AND colonoscopic:ab,ti) OR (surgery,:ab,ti AND colonoscopic:ab,ti) OR (surgical:ab,ti AND procedures,:ab,ti AND colonoscopic:ab,ti) OR (colonoscopic:ab,ti AND surger*:ab,ti) OR (surgeries,:ab,ti AND colonoscopic:ab,ti) OR 'colonoscopy'/exp

#5. 'colonoscopy'/exp

#4. colonoscop*:ab,ti OR (colonoscopic:ab,ti AND surgical:ab,ti AND procedure*:ab,ti) OR (procedure,:ab,ti AND colonoscopic:ab,ti AND surgical:ab,ti) OR (procedures,:ab,ti AND colonoscopic:ab,ti AND surgical:ab,ti) OR (surgical:ab,ti AND procedure,:ab,ti AND colonoscopic:ab,ti) OR (surgery,:ab,ti AND colonoscopic:ab,ti) OR (surgical:ab,ti AND

procedures,:ab,ti AND colonoscopic:ab,ti) OR (colonoscopic:ab,ti AND surger*:ab,ti) OR (surgeries,:ab,ti AND colonoscopic:ab,ti)

#3. polyethylene:ab,ti AND glycol*:ab,ti OR macrogol*:ab,ti OR (glycol,:ab,ti AND polyethylene:ab,ti) OR (glycols,:ab,ti AND polyethylene:ab,ti) OR (polyethylene:ab,ti AND oxide*:ab,ti) OR (oxide,:ab,ti AND polyethylene:ab,ti) OR (oxides,:ab,ti AND polyethylene:ab,ti) OR polyethyleneoxide*:ab,ti OR polyoxyethylene:ab,ti OR tritons:ab,ti OR 'macrogol derivative'/exp

#2. 'macrogol derivative'/exp

#1. polyethylene:ab,ti AND glycol*:ab,ti OR macrogol*:ab,ti OR (glycol,:ab,ti AND polyethylene:ab,ti) OR (glycols,:ab,ti AND polyethylene:ab,ti) OR (polyethylene:ab,ti AND oxide*:ab,ti) OR (oxide,:ab,ti AND polyethylene:ab,ti) OR (oxides,:ab,ti AND polyethylene:ab,ti) OR polyethyleneoxide*:ab,ti OR polyoxyethylene:ab,ti OR tritons:ab,ti
