# Supplementary material for: Comparative Efficacy of 2 L Polyethylene Glycol Alone or With Ascorbic Acid vs. 4 L Polyethylene Glycol for Colonoscopy: A Systematic Review and Network Meta-Analysis of 12 Randomized Controlled Trials
Source: Front Med (Lausanne). 2019 Aug 21;6:182. doi: 10.3389/fmed.2019.00182 (PMC6713044; doi:10.3389/fmed.2019.00182)
Supplement: Supplementary file 2 [file Data_Sheet_1.PDF]

Appendix

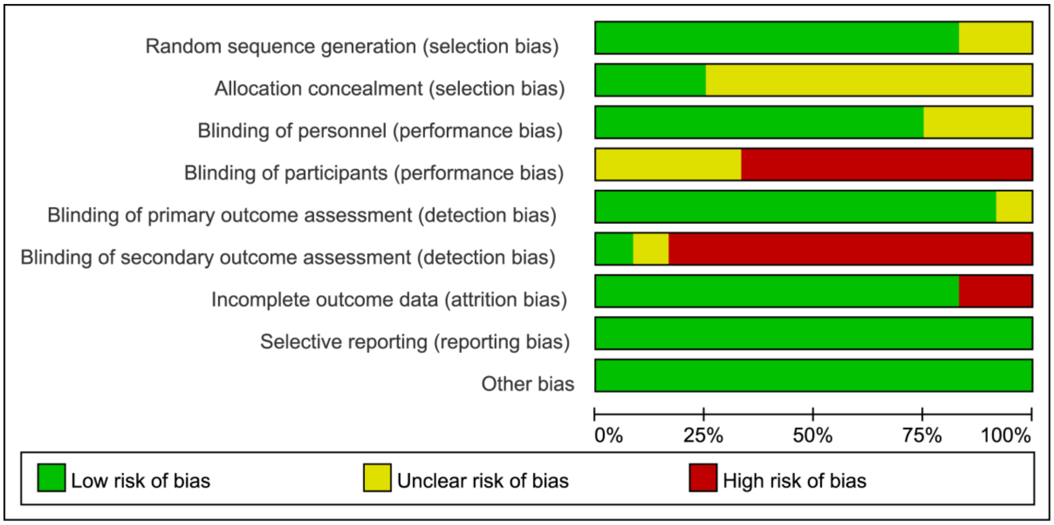

Figure S1: The cumulative percentages graph for each risk of bias domain. The yellow, green and red represent "unclear risk of bias", "low risk of bias" and "high risk of bias" respectively. The wide of a color indicated percentages of risk of bias.

	Random sequence generation (selection bias)	Allocation concealment (selection bias)	Blinding of personnel (performance bias)	Blinding of participants (performance bias)	Blinding of primary outcome assessment (detection bias)	Blinding of secondary outcome assessment (detection bias)	Incomplete outcome data (attrition bias)	Selective reporting (reporting bias)	Other bias
Ell C 2008	+	+	+	?	+	−	+	+	+
Jung YS 2016	+	?	+	−	+	−	+	+	+
Kanie H 2016	+	?	+	−	+	−	+	+	+
Kim Ms 2016	+	?	+	−	+	−	−	+	+
Lee BC 2008	+	?	+	−	+	−	+	+	+
Marmo R 2010	+	+	+	−	+	−	+	+	+
Moon CM 2014	?	?	?	?	+	−	+	+	+
Paggi S 2015	+	?	?	?	?	?	+	+	+
Park D 2013	?	?	+	−	+	−	+	+	+
Ponchon T 2013	+	?	+	?	+	−	+	+	+
Rivas JM 2014	+	?	+	−	+	+	−	+	+
Valiante F 2012	+	+	?	−	+	−	+	+	+

Figure S2: Risk of bias summary for individual randomized controlled trials. The yellow (question mark), green (plus sign) and red (minus sign) represent "unclear risk of bias", "low risk of bias" and "high risk of bias" respectively.

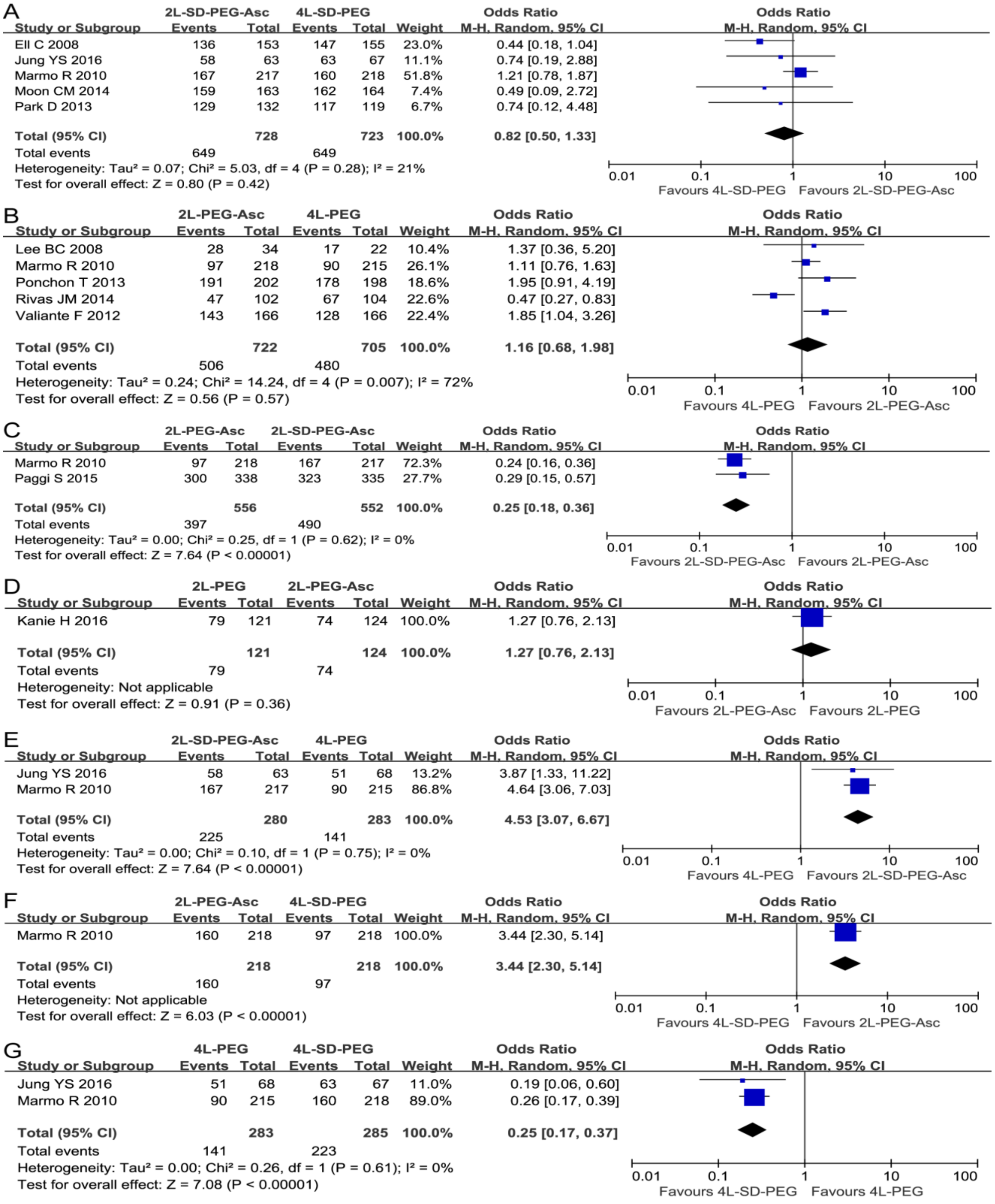

Figure S3: Forest plot for the bowel preparation efficacy of all direct comparisons. The summary effect estimate (odds ratio, OR) for individual randomized controlled trials (RCTs) are indicated by blue rectangles (the size of the rectangle is proportional to the study weight), with the black horizontal lines representing 95% confidence intervals (CIs). The overall summary effect estimate (OR) and 95% confidence interval are indicated by the black diamond below.

Study or Subgroup	2L-SD-PEG-Asc Events	2L-SD-PEG-Asc Total	4L-SD-PEG Events	4L-SD-PEG Total	Weight	Odds Ratio M-H, Random, 95% CI
**1.3.1 Compliance**						
Ell C 2008	130	153	135	155	52.0%	0.84 [0.44, 1.60]
Jung YS 2016	61	63	62	67	7.7%	2.46 [0.46, 13.16]
Kim Ms 2016	154	159	154	160	14.9%	1.20 [0.36, 4.01]
Marmo R 2010	209	217	209	218	23.0%	1.13 [0.43, 2.97]
Moon CM 2014	163	163	160	164	2.5%	9.17 [0.49, 171.67]
**Subtotal (95% CI)**		**755**		**764**	**100.0%**	**1.09 [0.68, 1.74]**
Total events	717		720			
Heterogeneity: Tau² = 0.00; Chi² = 3.66, df = 4 (P = 0.45); I² = 0%						
Test for overall effect: Z = 0.37 (P = 0.71)						
**1.3.2 Preference to repeat the same regimen**						
Jung YS 2016	50	63	41	67	25.5%	2.44 [1.11, 5.34]
Kim Ms 2016	102	159	57	160	74.5%	3.23 [2.05, 5.11]
**Subtotal (95% CI)**		**222**		**227**	**100.0%**	**3.01 [2.03, 4.47]**
Total events	152		98			
Heterogeneity: Tau² = 0.00; Chi² = 0.37, df = 1 (P = 0.54); I² = 0%						
Test for overall effect: Z = 5.46 (P < 0.00001)						
**1.3.3 Acceptance**						
Ell C 2008	113	153	82	155	35.7%	2.51 [1.56, 4.06]
Jung YS 2016	54	63	51	67	14.8%	1.88 [0.76, 4.64]
Kim Ms 2016	134	159	91	160	31.8%	4.06 [2.39, 6.90]
Moon CM 2014	155	163	130	164	17.7%	5.07 [2.27, 11.33]
**Subtotal (95% CI)**		**538**		**546**	**100.0%**	**3.18 [2.17, 4.66]**
Total events	456		354			
Heterogeneity: Tau² = 0.05; Chi² = 4.34, df = 3 (P = 0.23); I² = 31%						
Test for overall effect: Z = 5.91 (P < 0.00001)						
**1.3.4 Adverse events**						
Ell C 2008	73	153	86	155	28.7%	0.73 [0.47, 1.15]
Jung YS 2016	20	63	25	67	14.7%	0.78 [0.38, 1.61]
Kim Ms 2016	69	159	69	160	29.1%	1.01 [0.65, 1.57]
Moon CM 2014	94	163	120	164	27.5%	0.50 [0.31, 0.79]
**Subtotal (95% CI)**		**538**		**546**	**100.0%**	**0.73 [0.53, 1.00]**
Total events	256		300			
Heterogeneity: Tau² = 0.04; Chi² = 4.67, df = 3 (P = 0.20); I² = 36%						
Test for overall effect: Z = 1.97 (P = 0.05)						

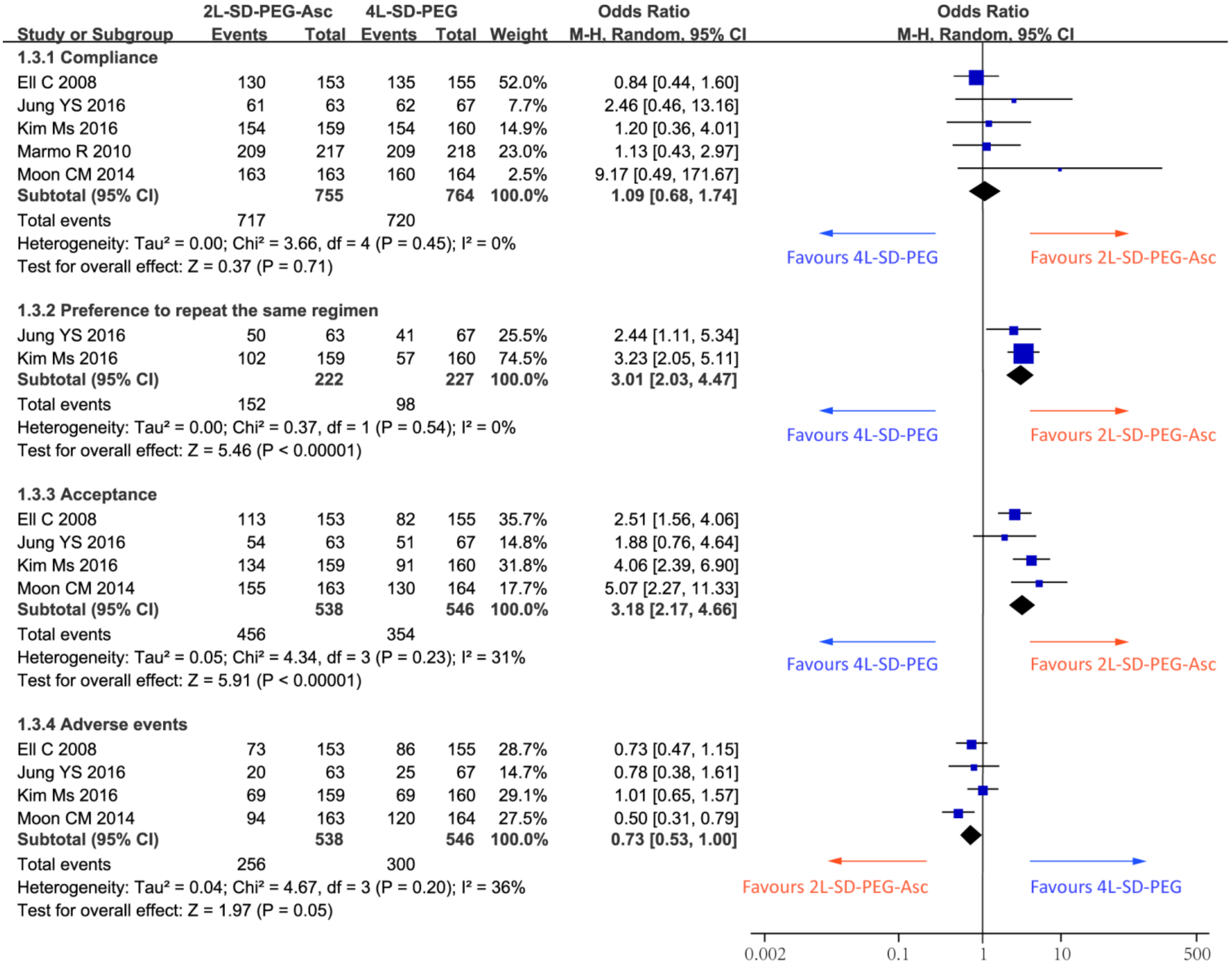

Figure S4: Forest plot for the secondary outcomes of 2L-SD-PEG-Asc versus 4L-SD-PEG. The summary effect estimate (odds ratio, OR) for individual randomized controlled trials (RCTs) are indicated by blue rectangles (the size of the rectangle is proportional to the study weight), with the black horizontal lines representing 95% confidence intervals (CIs). The overall summary effect estimate (OR) and 95% confidence interval are indicated by the black diamond below.

	2L-SD-PEG-Asc		4L-PEG			Odds Ratio
Study or Subgroup	Events	Total	Events	Total	Weight	M-H, Fixed, 95% CI
**5.4.1 Compliance**						
Jung YS 2016	61	63	41	68	13.8%	20.09 [4.53, 89.11]
Marmo R 2010	209	217	212	215	86.2%	0.37 [0.10, 1.41]
**Subtotal (95% CI)**		**280**		**283**	**100.0%**	**3.08 [1.51, 6.30]**
Total events	270		253			
Heterogeneity: Chi² = 15.69, df = 1 (P < 0.0001); I² = 94%						
Test for overall effect: Z = 3.09 (P = 0.002)						
**5.4.2 Preference**						
Jung YS 2016	50	63	43	68	100.0%	2.24 [1.02, 4.90]
**Subtotal (95% CI)**		**63**		**68**	**100.0%**	**2.24 [1.02, 4.90]**
Total events	50		43			
Heterogeneity: Not applicable						
Test for overall effect: Z = 2.01 (P = 0.04)						
**5.4.3 Acceptance**						
Jung YS 2016	54	63	49	68	100.0%	2.33 [0.96, 5.62]
**Subtotal (95% CI)**		**63**		**68**	**100.0%**	**2.33 [0.96, 5.62]**
Total events	54		49			
Heterogeneity: Not applicable						
Test for overall effect: Z = 1.88 (P = 0.06)						
**5.4.4 Adverse events**						
Jung YS 2016	20	63	31	68	100.0%	0.56 [0.27, 1.13]
**Subtotal (95% CI)**		**63**		**68**	**100.0%**	**0.56 [0.27, 1.13]**
Total events	20		31			
Heterogeneity: Not applicable						
Test for overall effect: Z = 1.62 (P = 0.11)						

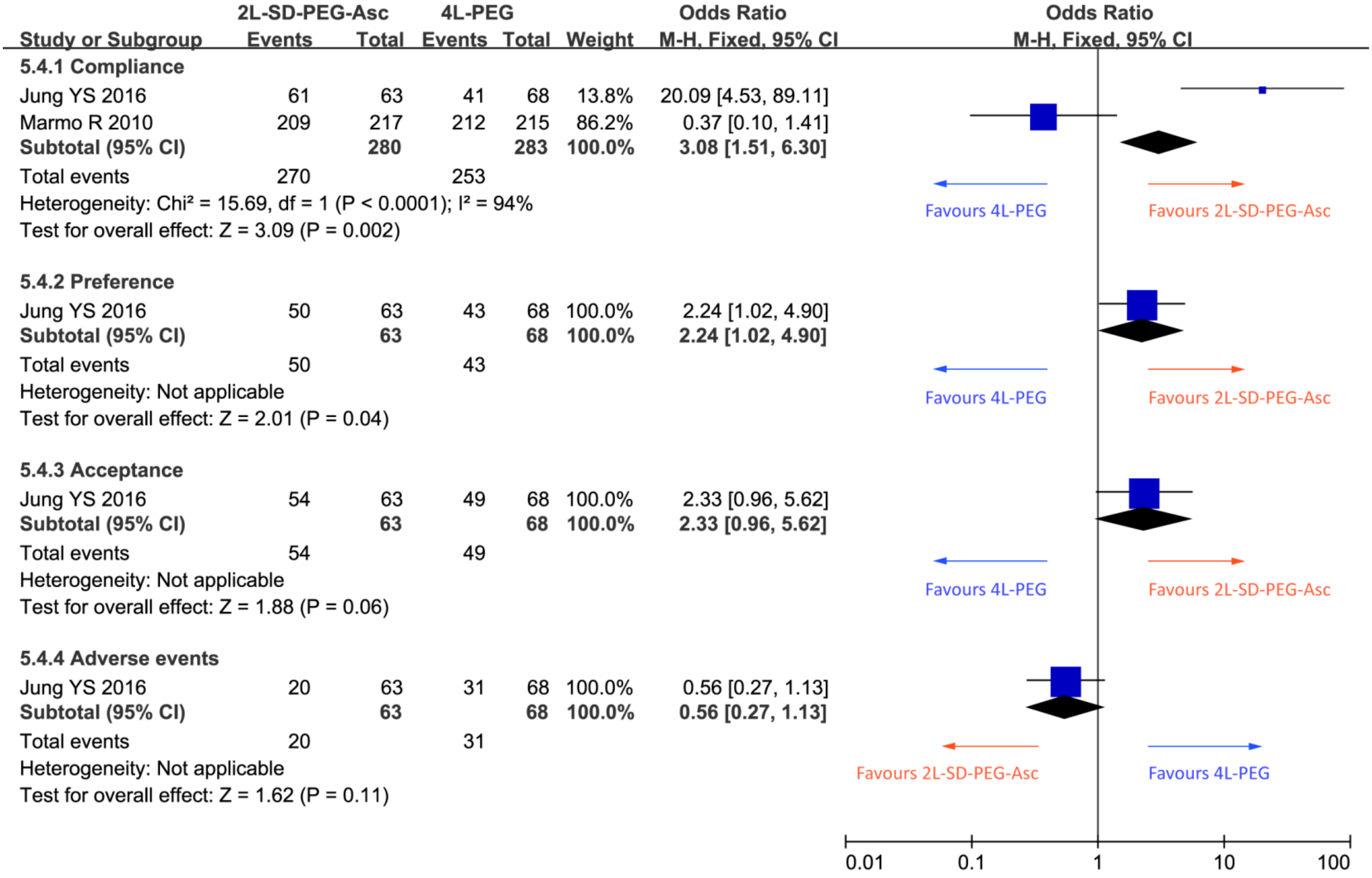

Figure S5: Forest plot for the secondary outcomes of 2L-SD-PEG-Asc versus 4L -PEG. The summary effect estimate (odds ratio, OR) for individual randomized controlled trials (RCTs) are indicated by blue rectangles (the size of the rectangle is proportional to the study weight), with the black horizontal lines representing 95% confidence intervals (CIs). The overall summary effect estimate (OR) and 95% confidence interval are indicated by the black diamond below.

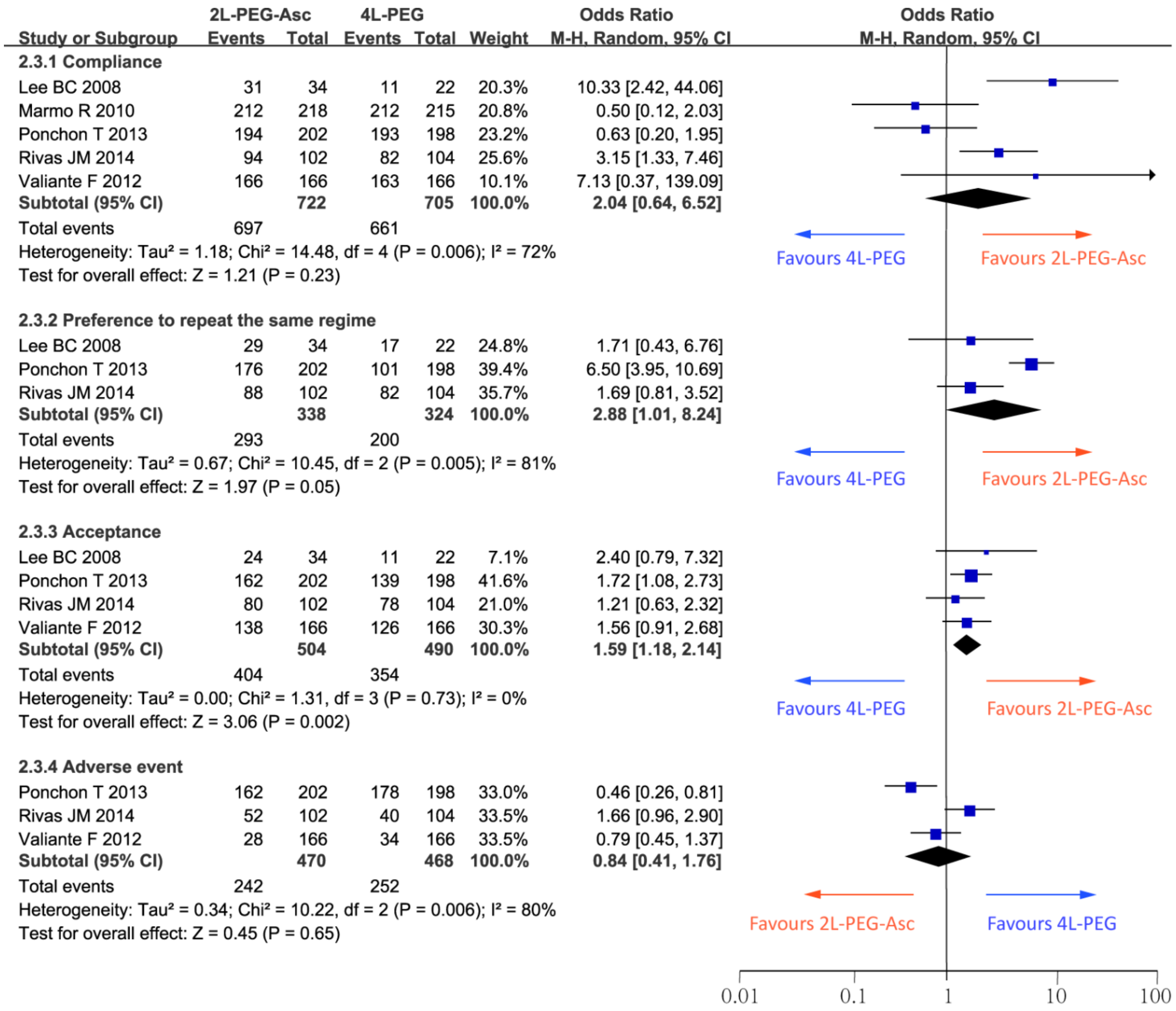

Figure S6: Forest plot for the secondary outcomes of 2L-PEG-Asc versus 4L-PEG. The summary effect estimate (odds ratio, OR) for individual randomized controlled trials (RCTs) are indicated by blue rectangles (the size of the rectangle is proportional to the study weight), with the black horizontal lines representing 95% confidence intervals (CIs). The overall summary effect estimate (OR) and 95% confidence interval are indicated by the black diamond below.

Study or Subgroup	2L-PEG-Asc Events	2L-PEG-Asc Total	4L-SD-PEG Events	4L-SD-PEG Total	Weight	Odds Ratio M-H, Random, 95% CI
Marmo R 2010	212	218	209	218	100.0%	1.52 [0.53, 4.35]
**Total (95% CI)**		**218**		**218**	**100.0%**	**1.52 [0.53, 4.35]**
Total events	212		209			
Heterogeneity: Not applicable						
Test for overall effect: Z = 0.78 (P = 0.43)						

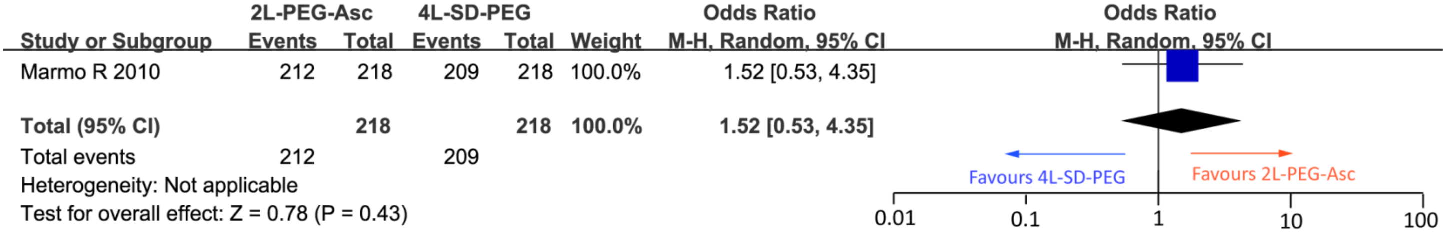

Figure S7: Forest plot for the compliance with recommend regime of 2L-PEG-Asc versus 4L-SD-PEG. The summary effect estimate (odds ratio, OR) for individual randomized controlled trials (RCTs) are indicated by blue rectangles (the size of the rectangle is proportional to the study weight), with the black horizontal lines representing 95% confidence intervals (CIs). The overall summary effect estimate (OR) and 95% confidence interval are indicated by the black diamond below.

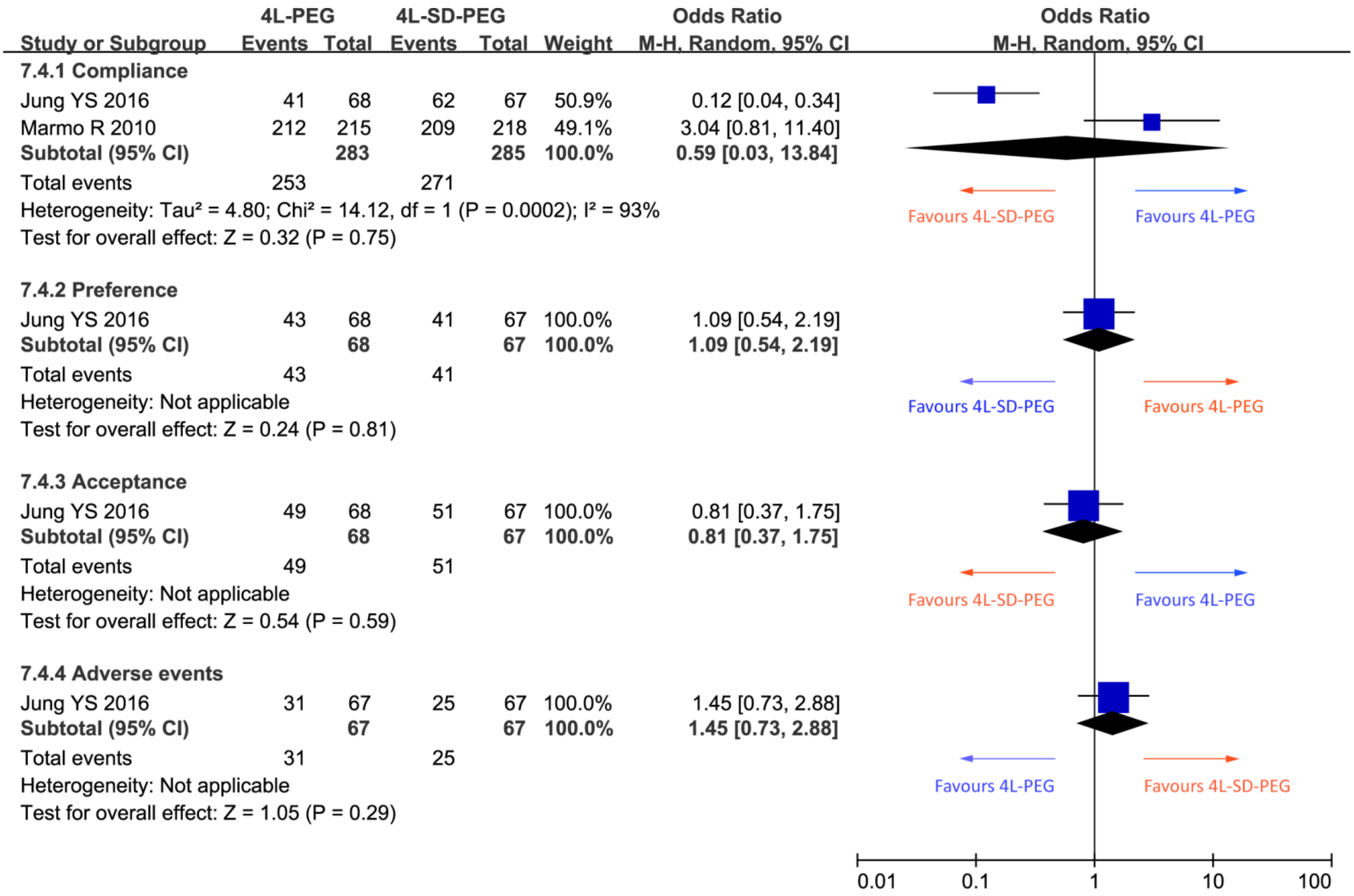

Figure S8: Forest plot for the secondary outcomes of 4L-PEG versus 4L-SD-PEG. The summary effect estimate (odds ratio, OR) for individual randomized controlled trials (RCTs) are indicated by blue rectangles (the size of the rectangle is proportional to the study weight), with the black horizontal lines representing 95% confidence intervals (CIs). The overall summary effect estimate (OR) and 95% confidence interval are indicated by the black diamond below.

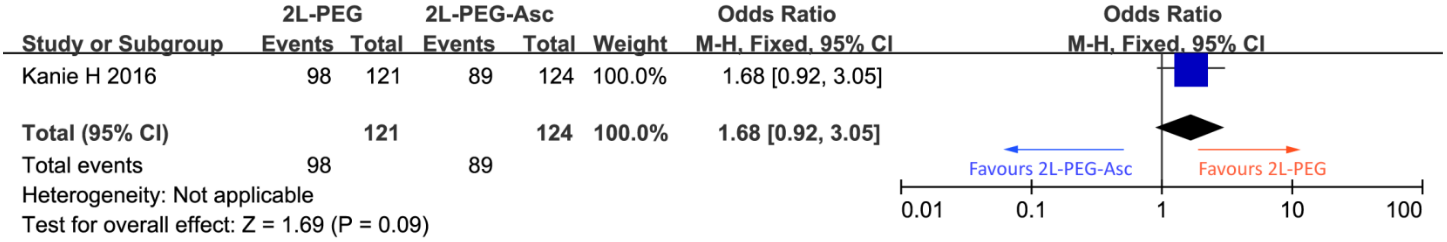

Figure S9: Forest plot for the acceptance to regime of 2L-PEG versus 2L-PEG-Asc. The summary effect estimate (odds ratio, OR) for individual randomized controlled trials (RCTs) are indicated by blue rectangles (the size of the rectangle is proportional to the study weight), with the black horizontal lines representing 95% confidence intervals (CIs). The overall summary effect estimate (OR) and 95% confidence interval are indicated by the black diamond below.

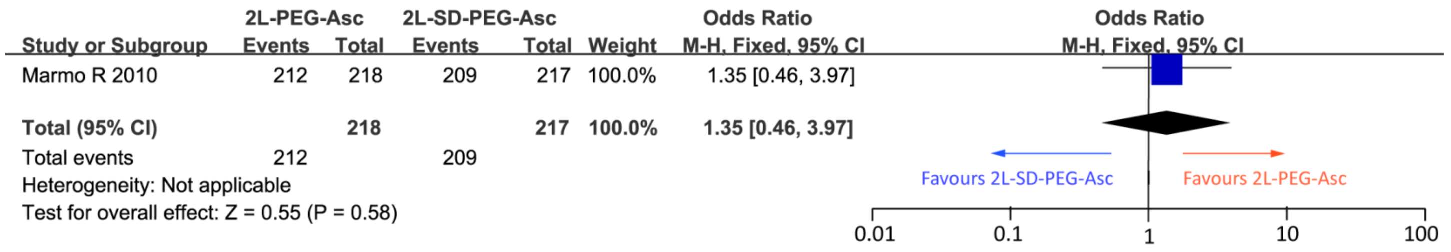

Figure S10: Forest plot for the compliance with recommend regime of 2L-PEG-Asc versus 2L-SD-PEG-Asc. The summary effect estimate (odds ratio, OR) for individual randomized controlled trials (RCTs) are indicated by blue rectangles (the size of the rectangle is proportional to the study weight), with the black horizontal lines representing 95% confidence intervals (CIs). The overall summary effect estimate (OR) and 95% confidence interval are indicated by the black diamond below.

A

2L-PEG-Asc	1.35 (0.46, 3.97)	2.04 (0.64, 6.52)	1.52 (0.53, 4.35)
0.87 (0.07, 10.22)	2L-SD-PEG-Asc	**3.08 (1.51, 6.30)**	1.09 (0.68, 1.74)
3.32 (0.65, 19.73)	3.73 (0.46, 37.40)	4L-PEG	**0.43 (0.22, 0.82)**
1.62 (0.16, 22.97)	1.87 (0.38, 11.49)	0.49 (0.06, 4.72)	4L-SD-PEG

B

2L-PEG-Asc	n.a.	**2.88 (1.01, 8.24)**	n.a.
1.25 (0.11, 10.75)	2L-SD-PEG-Asc	**2.24 (1.02, 4.90)**	**3.01 (2.03, 4.47)**
3.11 (0.86, 9.78)	2.52 (0.36, 18.91)	4L-PEG	1.09 (0.54, 2.19)
3.68 (0.32, 31.14)	2.92 (0.69, 12.13)	1.15 (0.16, 7.68)	4L-SD-PEG

C

2L-PEG	1.68 (0.92, 3.05)	n.a.	n.a.	n.a.
1.61 (0.69, 3.89)	2L-PEG-Asc	n.a.	**1.59 (1.18, 2.14)**	n.a.
0.83 (0.22, 3.28)	0.51 (0.17, 1.50)	2L-SD-PEG-Asc	2.33 (0.96, 5.62)	**3.18 (2.17, 4.66)**
**2.59 (1.01, 7.21)**	**1.59 (1.03, 2.50)**	**3.15 (1.22, 8.44)**	4L-PEG	0.81 (0.37, 1.75)
2.69 (0.73, 10.29)	1.65 (0.58, 4.73)	**3.23 (2.06, 5.07)**	1.02 (0.40, 2.66)	4L-SD-PEG

D

2L-PEG-Asc	n.a.	0.84 (0.41, 1.76)	n.a.
1.58 (0.47, 5.34)	2L-SD-PEG-Asc	0.56 (0.27, 1.13)	0.73 (0.53, 1.00)
0.85 (0.45, 1.56)	0.53 (0.19, 1.53)	4L-PEG	1.41 (0.71, 2.80)
1.15 (0.35, 3.88)	0.73 (0.43, 1.25)	1.36 (0.49, 3.79)	4L-SD-PEG

Figure S11: Summary for CP (A), PRSR (B), AT (C), and AEs (D) of different PEG-based bowel preparation regimes. The upper right area represented the effect sizes of direct comparisons and the bottom left shown the network comparisons. For direct comparison, it favors the row-defining treatment if odds ratio (OR) lower than 1, in contrast, for indirect comparison, the result favors the column-defining treatment if OR lower than 1. For numerical data, each number in each cell represented the effect size of the treatment in upper left area minus the treatment in bottom right area. Bold font represented statistical significance. PEG = polyethylene glycol, Asc = ascorbic acid, SD = split-dose, n.a. = not available.

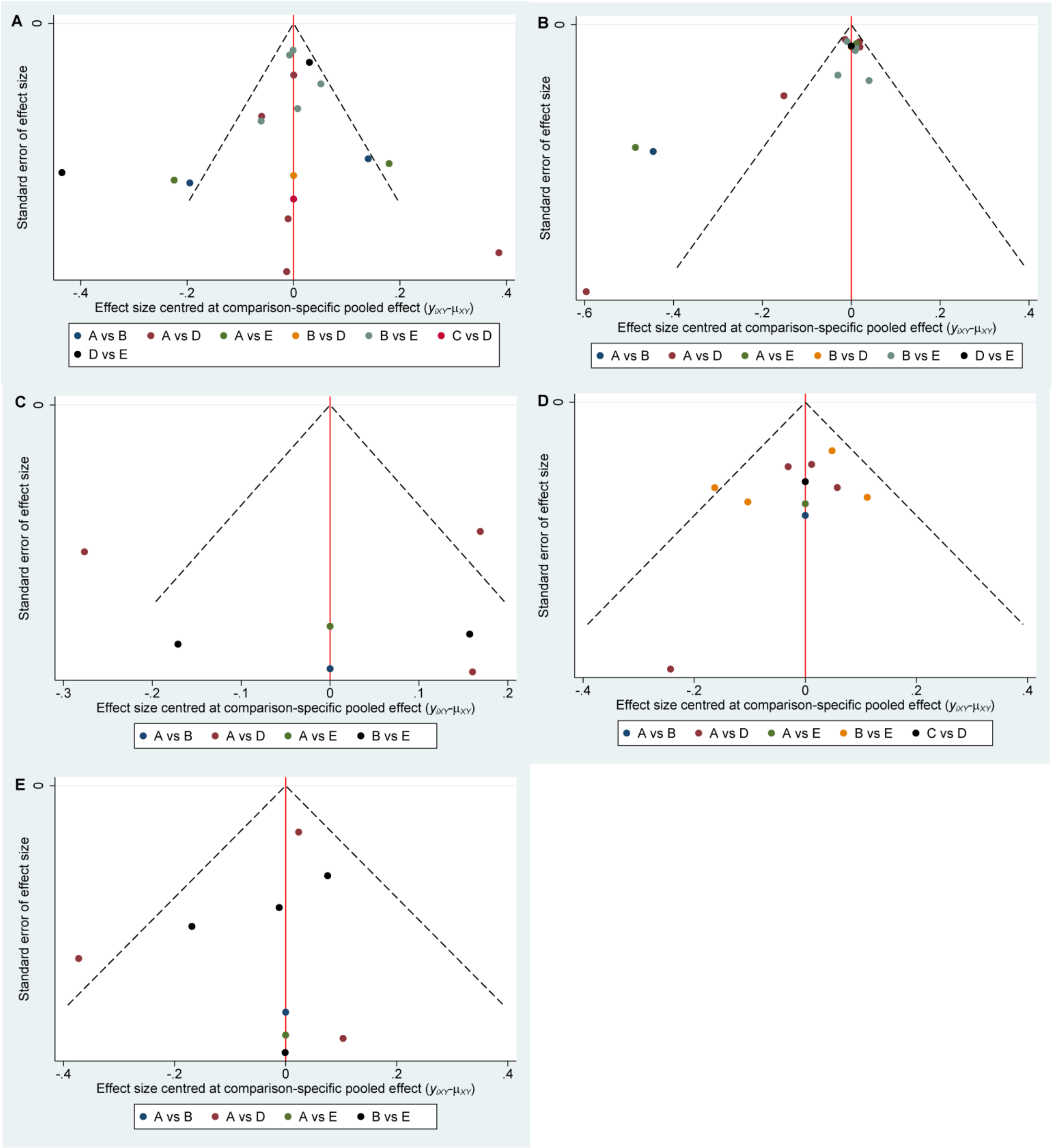

Figure S12: Comparison-adjusted funnel for bowel preparation efficacy (A), Compliance with recommend regime (B), preference to repeat the same regime (C), acceptance to regime (D), and adverse events (E). The vertical axis represented the standard error (SE) of effect size and x axis indicated the difference between effect size and pooled effect. Asymmetrical funnel plot indicated small study effect. The A represented 4L-PEG, B represented 4L-SD-PEG, C represented 2L-PEG, D represents 2L-PEG-Asc, and D represented 2L-SD-PEG-Asc.
